# Mechanical Properties and Elastic Modulus Prediction of Mixed Coal Gangue Concrete

**DOI:** 10.3390/ma18061240

**Published:** 2025-03-11

**Authors:** Xipeng Qin, Zhengyi Xu, Mingyu Liu, Yingying Zhang, Yixiang Wang, Zhongnian Yang, Xianzhang Ling

**Affiliations:** 1 School of Civil Engineering, Harbin Institute of Technology, Harbin 150090, China; lingxianzhang@qut.edu.cn; 2 School of Civil Engineering, Qingdao University of Technology, Qingdao 266033, China; 1361649105@163.com (M.L.); zluyankee@163.com (Y.Z.); 18366156702@163.com (Y.W.); 3 School of Civil Engineering, Tianjin University, Tianjin 300072, China; 17852835318@163.com

**Keywords:** mixed coal gangue aggregate, concrete, mechanical properties, elastic modulus, predictive model

## Abstract

Coal gangue, representing an industrial waste with the highest annual emissions and largest cumulative stocks worldwide, urgently requires resource utilization. This article uses mixed coal gangue aggregates (spontaneous-combustion coal gangue aggregate (SCGA) and rock coal gangue aggregate (RCGA)) as the research subject. The aim is to solve the technical problem of producing high-performance concrete with gangue instead of coarse aggregate. The research investigates the impact of various strength grades (C20, C30, C40, C50) and aggregate replacement ratios (0%, 20%, 40%, 60%, 80%, 100%) on the compressive strength of concrete. It explores the mechanical behaviors and properties of concrete mixed with coal gangue and develops a predictive model for its elastic modulus. The results show that (1) as the substitution rate of aggregates increases, the elastic modulus and compressive strength of the mixed coal gangue concrete significantly decrease. When the substitution rate is 100%, the elastic modulus and compressive strength of C30 concrete decrease by 3.5% and 11.3%, respectively, and the higher the grade, the more significant the reduction. For C50 concrete, the elastic modulus and compressive strength decrease by 10% and 35%, respectively. (2) A regression equation has been formulated to delineate the relationship between the compressive strength and axial compressive strength of mixed coal gangue concrete, taking into account the mix ratio of coal gangue and the compressive strength of standard concrete. This equation elucidates the correlation between the mechanical properties of concrete with varying coal gangue mix ratios and ordinary concrete across different strength grades. (3) Based on the correlation between elastic modulus and compressive strength, a prediction model for the elastic modulus of mixed gangue concrete was established, which effectively improved its prediction accuracy.

## 1. Introduction

Coal gangue is an industrial solid waste generated during the mining, processing, and power generation of coal. It ranks among the largest industrial solid wastes in China, constituting about 25% of the nation’s industrial waste emissions [1] and roughly 10% of coal production [2], with an annual output ranging from approximately 500 to 800 million tons [3]. Abandoned coal gangue consumes land resources and contaminates the air, soil, and groundwater [4,5]. Furthermore, coal gangue comprises residual coal, carbonaceous shale, wood fragments, and other combustibles, which may result in spontaneous combustion when accumulated outdoors for extended periods [4]. Approximately one-third of China’s over 2300 gangue hills have encountered such incidents. The effective and comprehensive utilization of coal gangue has emerged as a pressing issue requiring resolution.

Concrete is the most extensively utilized building material, with a substantial overall consumption, and aggregates constitute approximately 70–80% of its volume [6]. Global annual concrete consumption reaches about 17.5 billion tons, with aggregate use surpassing 1.3 billion tons [7], notably river sand and natural gravel, which are non-renewable resources. Additionally, the extraction process results in significant environmental pollution. The primary constituents of coal gangue, SiO_2_ and Al_2_O_3_, are akin to those found in river sand and natural gravel. Consequently, employing coal gangue in concrete production is a significant step towards enhancing its utilization and mitigating the scarcity of natural aggregates. In addition, it can reduce the purchase of aggregate and improve economic efficiency.

Coal gangue can be integrated into concrete through three methods: (1) substituting a portion of the fine aggregate [8,9], entirely replacing the coarse aggregate [10,11,12,13,14], and (2) wholly replacing both coarse and fine aggregates [15,16]. Currently, coal gangue aggregate concrete is not extensively utilized in building structures. While the fundamental structure of coal gangue resembles that of conventional sand and stone aggregates, the mechanical performance indicators vary significantly due to factors like region, source, and type. The microstructure lacks density, and initial defects, such as a complex pore structure, are more prevalent than in ordinary sand and gravel aggregates, leading to greater variability in the macroscopic mechanical properties of coal gangue aggregate concrete. On the other hand, the absence of clear technical specifications for coal gangue as a concrete aggregate, alongside limited industry regulations for SCGA used as a lightweight aggregate, has led to a bottleneck in the widespread application and promotion of RCGA concrete, impacting its structural design specifications and application scope. In recent years, there has been considerable research on the mechanical properties of RCGA concrete. For instance, Liu et al. [17] discovered that the substitution rate of coal gangue significantly influences the mechanical properties of concrete. The elastic modulus of concrete using 100% SCGA and 100% RCGA aggregates decreased by 57% and 41%, respectively. Wang et al. [18] discovered that the RCGA content significantly influences concrete’s compressive strength, sulfate resistance, and freeze resistance. Xiao et al. [19] discovered that larger coarse aggregate sizes enhance the compressive strength of concrete. Coal gangue concrete with comprehensive particle size grading exhibits the highest compressive strength, while the grading of aggregates has a minimal impact on compressive strength. Nonetheless, research on the mechanical properties of mixed coal gangue aggregate concrete is limited, failing to adequately convey its mechanical characteristics.

This study utilizes mixed coal gangue to substitute natural gravel in varying proportions of 0%, 20%, 40%, 60%, 80%, and 100% to formulate coal gangue concrete with design strength grades of C20, C30, C40, and C50. Initially, by examining the variations and interconnections in macroscopic properties like compressive strength, axial compressive strength, and elastic modulus of concrete without admixtures, regression equations were formulated to describe the relationship between mixed coal gangue content and both cube compressive strength and axial compressive strength. Secondly, by analyzing the relationship between the compressive strength and elastic modulus of recycled concrete from various studies, a predictive model for the elastic modulus of coal gangue concrete was developed. Finally, the influence of coal gangue on the mechanical properties of concrete is explained, and the interaction mechanism between mixed coal gangue aggregate and the cementitious matrix is revealed, offering a theoretical foundation for predicting the mechanical traits and elastic modulus of mixed coal gangue concrete in large-scale applications.

## 2. Materials and Methods

### 2.1. Materials

The experiment uses P.O42.5R ordinary Portland cement, which was produced by the Jidong Company of Tangshan City, Hebei Province, China, and the cement indexes met the quality requirements of China GB175-2007 “General Portland Cement”. Polycarboxylic acid series (TPEG) high-performance water-reducing agent was used as the water-reducing agent, produced by Shenyang Ylida Building Admixture Factory, Shenyang, China. Coal gangue, as a substitute for coarse aggregate gravel, has the same various indicators as gravel. The coal gangue produced by China Chaoyang Hualong Building Materials Co., Ltd. (Beijing, China) in the same area is selected, which comprises a blend of SCGA and RCGA to eliminate any experimental discrepancies arising from variations in the coal gangue composition. According to the requirements of GB/T14685-2011 “Pebbles and gravel for construction”, the main chemical composition and mineral composition of mixed gangue were analyzed, and the basic properties of mixed gangue aggregate were determined. The mineral composition, chemical composition, and technical properties are presented in Table 1, Table 2 and Table 3, respectively. The grading of coarse aggregate is shown in Table 4 [20].

### 2.2. Mixed Coal Gangue Concrete Mix Design

The formulation of the concrete mix ratio adheres to the specifications outlined in JGJ55-2011, “Ordinary Concrete Mix Proportion Design Code”. Ordinary crushed stone is employed as the coarse aggregate, with design strength grades of C20, C30, C40, and C50. The slump is set at 180 ± 20 mm. Then, concrete was made using mixed coal gangue as a replacement for natural crushed stone at replacement levels of 0%, 20%, 40%, 60%, 80%, and 100%. The concrete composition was adjusted with admixtures, and specimens of mixed coal gangue concrete were prepared. Tests were conducted on the compressive strength, axial compressive strength, and elastic modulus of the mixed coal gangue concrete, and the effects of the mixed coal gangue content on the fluidity, compressive strength, axial compressive strength, and elastic modulus of the concrete were analyzed and studied. As the replacement rate of mixed coal gangue increases, the fluidity of the concrete decreases. To ensure the consistency of the concrete, the amount of admixtures can be adjusted accordingly. After trial matching, the mix proportion of coal gangue concrete is shown in Table 5.

### 2.3. Test Method

(1) Compressive strength

According to the “Test Method for Mechanical Properties of Ordinary Concrete” (GB/T50081-2019), the compressive strength test of concrete specimens cured to 28 years old was carried out. The test specimen used in the cube compression test is a 100 × 100 × 100 mm cube specimen, and the instrument used in the test is a 1000 kN electro-hydraulic servo universal testing machine. The loading rate used in this study was 0.6 MPa/s, and three specimens were tested for each mix ratio.

(2) Axial compressive strength

According to the “Test Method for Mechanical Properties of Ordinary Concrete” (GB/T50081-2019), the axial compressive strength test of concrete specimens cured to 28 years old was carried out. The specimen size used in the axial compressive strength test is a 100 × 100 × 300 mm prismatic specimen, and the instrument used in the test is a 1000 kN electro-hydraulic servo universal testing machine. The loading rate of the axial compressive strength test is the same as that of the cube compressive strength, and the loading rate is 0.6 MPa/s within 60% of the estimated force. It was then loaded with a 0.5 mm/min displacement loading program until the specimen was broken and the loading was stopped; three specimens were tested for each mix ratio.

(3) Modulus of elasticity

According to the “Test Method for Mechanical Properties of Ordinary Concrete” (GB/T50081-2019), the elastic modulus test of concrete specimens cured to 28 years old was carried out. This test adopts the measuring method of a dial gauge. The specimens used are 100 mm × 100 mm × 300 mm prismatic specimens; each group has 3 specimens, and the loading speed is 1.0 MPa/s.

## 3. Analysis of Mechanical Properties of Mixed Coal Gangue Concrete Test Results

### 3.1. Mechanical Test of Mixed Coal Gangue Concrete

The coarse aggregate concrete mixed with coal gangue is prepared according to Table 5. The compressive strength, axial compressive strength, and modulus of elasticity after 28 days of curing are shown in Table 6.

**Figure 1:**
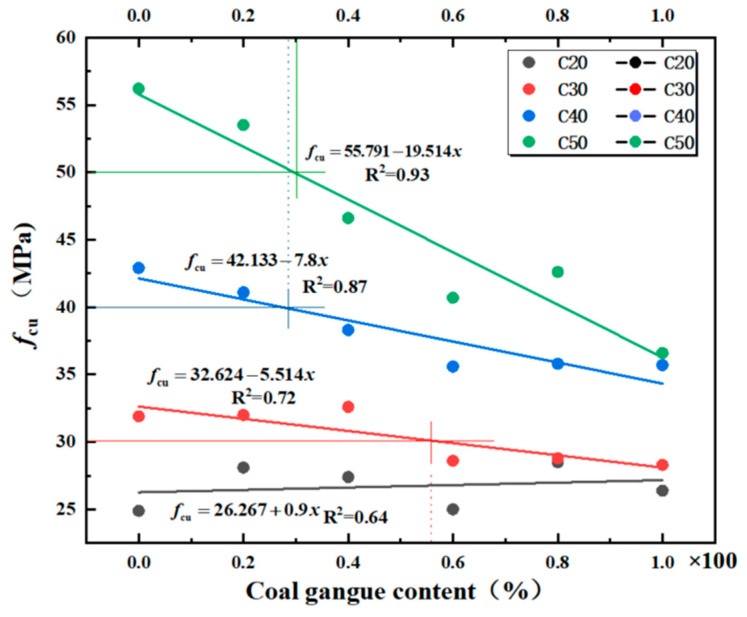
Regression Analysis Chart of Compressive Strength of Coal Gangue Concrete.

To better explain the relationship between the compressive strength of mixed coal gangue concrete cubes and the coal gangue content, a regression line of the cube compressive strength and coal gangue content relationship was established, as shown in Figure 1.

According to Table 6 and Figure 1, it is known that when mixed coal gangue substitutes for coarse aggregate in the preparation of C20 strength concrete, the increase in the amount of mixed coal gangue has little effect on the strength of the concrete. Incorporating mixed coal gangue to substitute up to 40% of coarse aggregate in C30 strength grade concrete and up to 20% in C40 and C50 strength grades fulfills the engineering specifications. Thus, when manufacturing concrete of grade C30 or higher using mixed coal gangue, the inclusion of such materials must be stringently controlled. Conversely, for lower-grade concrete, there are no limitations on the use of mixed coal gangue. This is attributed to RCGA’s high water absorption capacity [21], potentially lowering the water-to-cement ratio, whereas the volcanic activation of SCGA can enhance hydration reactions, consequently strengthening the interface and increasing the overall strength. During the strength development process of concrete, the moisture absorbed by RCGA is gradually released in the later stages, ensuring a good level of humidity inside the concrete. This provides the necessary moisture for the hydration of cementitious materials in the concrete. The hydration reaction continues, leading to further increases in the concrete’s strength over time, ensuring that the strength of C20 concrete remains constant or even slightly increases. Therefore, variations in the content of mixed coal gangue in C20 grade concrete have little impact on its strength and may even enhance it. When the mixing amount of coal gangue is within 40%, the strength of C30 concrete does not change significantly, but when the mixing amount increases to 60%, the strength shows a clear downward trend. More than half of the total mass of aggregates at this stage relies on the quality of mixed coal gangue. On the one hand, the crushing index of mixed coal gangue is low [22,23], and on the other hand, the dust on the surface of mixed coal gangue significantly affects the cement paste bonding [21,24], resulting in a mismatch between the strength of coarse aggregates and the cement matrix strength. The incorporation of mixed coal gangue significantly affects the failure process, failure mode, compressive strength, and structure of the concrete [25]. This phenomenon is particularly evident in C40 and C50 concrete. When the blending amount of mixed gangue exceeds 40%, the compressive strength of C40 and C50 concrete decreases by 11% and 17%, respectively. When the blending amount reaches 100%, the strength of C50 concrete decreases by 35%, which is much lower than the design strength.

### 3.2. The Relationship Between the Axial Compressive Strength of Mixed Coal Gangue Concrete and the Compressive Strength of Ordinary Concrete and the Coal Gangue Content

**Figure 2:**
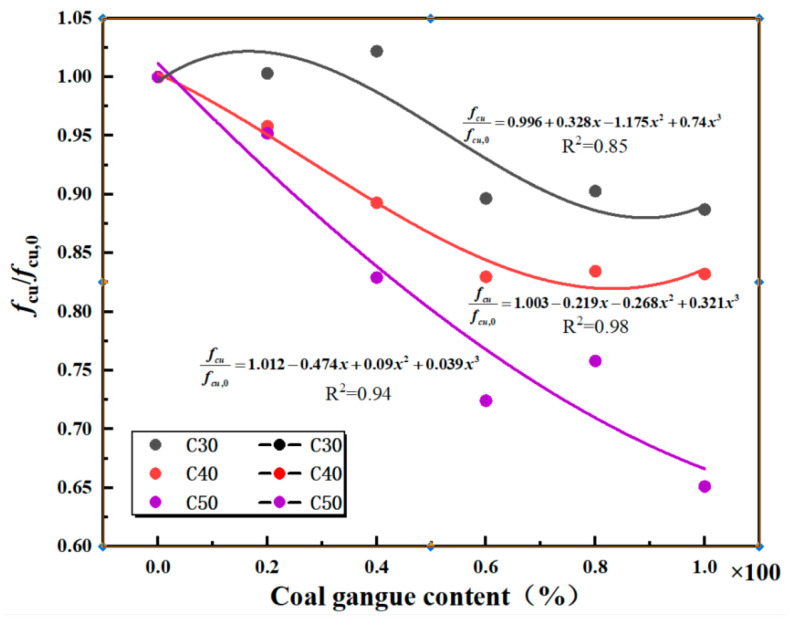
Gangue concrete compressive strength/axial compressive strength regression analysis plot.

Linear regression analysis was conducted on the compressive strength of mixed coal gangue concrete cubes (Figure 2), establishing a relationship with the compressive strength of ordinary concrete cubes. Based on the relationship between the mixed coal gangue content and the compressive strength of cubes, the relationship formulas for coal gangue concrete and ordinary concrete of strength grades C30, C40, and C50 are proposed as follows:

(1) $$\frac{f_{cu}}{f_{cu,0}}=a+bx+cx^{2}+dx^{3}$$

In the formula, *f_cu_*_,0_—cubic compressive strength corresponding to ordinary concrete (MPa); *x*—amount of mixed coal gangue added.

The results of the experiment and the linear regression are shown in Figure 2, from which the regression line Equation (2) can be derived.

(2) $$\frac{f_{cu}}{f_{cu,0}}=\left\{\begin{array}{ll} 0.996+0.328x-1.175x^{2}+0.74x^{3} & C30\\ 1.003-0.219x-0.268x^{2}+0.321x^{3} & C40\\ 1.012-0.474x+0.09x^{2}+0.039x^{3} & C50 \end{array}\right.$$

The known ratio of axial compressive strength to cubic compressive strength of ordinary concrete is 0.72–0.92. The standard given by China regarding the reliability of structures (GB 50068-2018, Unified Standard for Reliability Design of Building Structures) is *f_c_* = 0.76*f_cu_*. To explore the relationship between coal gangue concrete, see the calculation results in Table 7.

**Figure 3:**
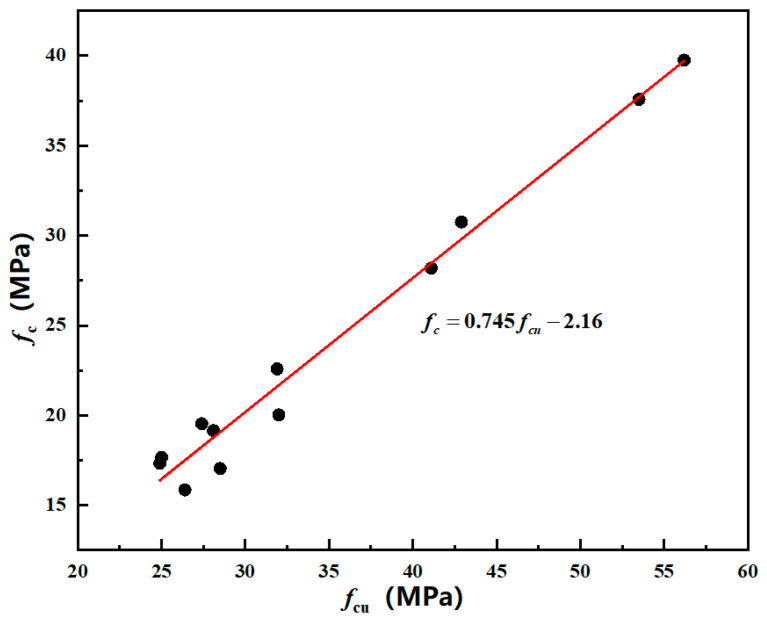
Regression analysis graph of coal gangue concrete *f_c_*, *f_cu_*.

According to the standard of normal concrete axial compressive strength, an analysis of the axial compressive strength results of mixed coal gangue concrete was conducted. It was found that the mixed coal gangue concrete prepared with strength C20 met the requirements (GB 50068-2018, Unified Standard for Reliability Design of Building Structures). Concrete with strength grades C30, C40, and C50, where mixed coal gangue replaced coarse aggregate by 20% or less, also met the requirements. A linear regression analysis was performed on the mixed coal gangue concrete *f_c_* and *f_cu_* that met the standards, and the results (Figure 3) are as follows:

(3) $$f_{c}=0.745f_{cu}-2.16$$

In the formula, *f_cu_* represents the cubic compressive strength of coal gangue concrete (MPa); *f_c_* represents the axial compressive strength of coal gangue concrete (MPa).

According to the results of linear regression analysis, the relationship formula between mixed coal gangue concrete *f_c_* and *f_cu_* is *f_c_* = 0.745*f_cu_* − 2.16, which is applicable to concrete strength grades C20 and coal gangue replacing coarse aggregate by 20% and below for strength grades C30, C40, C50 concrete. By substituting the expression of mixed coal gangue concrete, ordinary concrete, and coal gangue content from Formula (2) into Formula (3), the expression (4) for the axial compressive strength of mixed coal gangue concrete, ordinary concrete, and coal gangue content can be obtained.

(4) $$f_{c}=\left\{\begin{array}{ll} 0.742f_{cu,0}+0.244xf_{cu,0}-0.875x^{2}f_{cu,0}+0.551x^{3}f_{cu,0}-2.16 & C30\\ 0.747f_{cu,0}-0.163xf_{cu,0}-0.200x^{2}f_{cu,0}+0.239x^{3}f_{cu,0}-2.16 & C40\\ 0.754f_{cu,0}-0.353xf_{cu,0}+0.067x^{2}f_{cu,0}+0.030x^{3}f_{cu,0}-2.16 & C50 \end{array}\right.$$

In the formula, *f_cu_*_,0_—cubic compressive strength of ordinary concrete (MPa); *f_c_*—axial compressive strength of coal gangue concrete (MPa); *x*—mixing amount of coal gangue.

### 3.3. The Relationship Between Coal Gangue Content and Elastic Modulus

**Figure 4:**
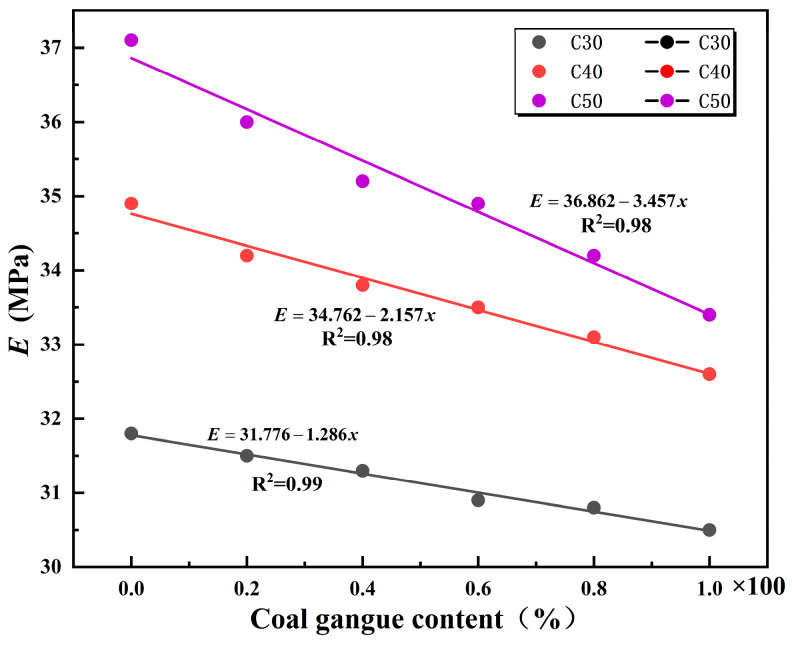
Regression Analysis Graph of Elastic Modulus of Mixed Coal Gangue Concrete.

To investigate the effect of coal gangue content on the modulus of elasticity, a relationship between the strength of mixed coal gangue concrete and the modulus of elasticity was established (Equation (5)), and the fitting curve is shown in Figure 4.

(5) $$E=\left\{\begin{array}{ll} 31.776-1.286x & C30\\ 34.762-2.157x & C40\\ 36.862-3.457x & C50 \end{array}\right.$$

In the formula, *E*—elastic modulus of mixed coal gangue concrete (GPa); *x*—content of mixed coal gangue.

Aggregates in concrete account for about 60~70% of the total volume of the concrete mixture, and the rock type, elastic modulus, shape, and surface structure of the aggregates have varying degrees of influence on the elastic modulus of the concrete [26]. The strength of the mixed coal gangue aggregate in the experiment is low, the surface structure is not dense, and its water absorption rate is higher than that of ordinary gravel. It also contains some impurities, which makes the performance of mixed coal gangue aggregate lower than that of gravel. Therefore, the properties of mixed coal gangue aggregate will significantly affect the elastic modulus of concrete.

As shown in Figure 4, as the concrete grade increases, the slope becomes steeper, indicating that the impact of mixed coal gangue on coal gangue concrete increases with the increase of concrete grade and as the substitution rate of mixed coal gangue in the total aggregate increases, the mixed coal gangue begins to play a decisive role in determining the performance of concrete. In C20 grade concrete, as the mixing ratio of coal gangue increases from 0 to 100%, the elastic modulus of the concrete fluctuates within ±1 GPa. This is due to the presence of micropores in the mixed coal gangue aggregate, which can provide a buffering and mitigating effect [27]. The elastic modulus of C30 grade concrete gradually decreases as the proportion of mixed coal gangue increases, and when the coal gangue content reaches 100%, the elastic modulus decreases by 3.5%. For concrete with strength grades C40 and C50, the change in the modulus of elasticity is quite significant. It shows a noticeable decrease when the admixture content is between 40 and 60%, with a maximum reduction of up to 6%. When the substitution rate of mixed gangue is 100%, the modulus of elasticity decreases by 10%. As the load on the concrete intensifies, the deformation of the mixed coal gangue escalates rapidly, whereas the deformation of the colloid remains relatively minor, preventing them from functioning cohesively. This suggests that the strength of the mixed coal gangue is insufficient to support the evolving requirements of concrete strength, resulting in the compromise of the concrete support frame’s operational performance. Therefore, in practical application, the content of mixed coal gangue should be properly determined according to different grades of concrete so as not to affect the quality of the concrete. From the analysis of references [9,28,29,30], it is known that the water absorption rate of coal gangue is higher than that of ordinary sand and gravel, indicating that coal gangue has a porous microstructure. Chen et al. [29] posit that the porosity of aggregates dictates their stiffness, which in turn governs the strain capacity of aggregate-constrained cement. Denser aggregates exhibit a higher modulus of elasticity; consequently, incorporating mixed gangue diminishes the concrete’s modulus of elasticity.

## 4. Predictive Model for the Elastic Modulus of Mixed Coal Gangue Concrete

In the structural utilization of concrete, the modulus of elasticity is a crucial parameter influencing its limit state of usage. The measurement of the elastic modulus, characterized by high precision, extended testing periods, and numerous influencing factors, displays significant variability in results [31]. Fundamentally, a power function relationship exists between the modulus of elasticity and the compressive strength of ordinary concrete [32,33,34]. However, due to the unique formation process of coal gangue concrete, its mechanical properties exhibit significant variability, complicating the direct determination of the corresponding relationship between its compressive strength and modulus of elasticity. At the same time, coal gangue concrete is composed of various materials, and the composition, properties, and forming conditions of the materials have a significant impact on the performance of the concrete. Therefore, there is a considerable discrepancy in the formulas for calculating the modulus of elasticity [35].

**Figure 5:**
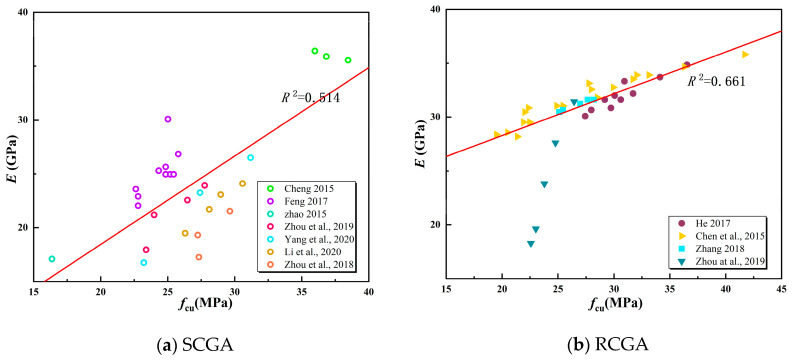
Regression analysis graph of elastic modulus and compressive strength of SCGA and RCGA concrete [29,36,37,38,39,40,41,42,43,44].

This research investigates the correlation between the elastic modulus and compressive strength using a combination of spontaneously combusting and non-spontaneously combusting coal gangue. The study reviews existing literature on SCGA (Cheng [36], Fang and Feng [37], Zhao [38], Zhou et al. [39,40], Yang et al. [41], Li et al. [42]) and RCGA (He [43], Chen et al. [29], Zhang [44], Zhou et al. [39]), as depicted in Figure 5, to analyze the relationship between the elastic modulus and compressive strength of mixed coal gangue.

**Figure 6:**
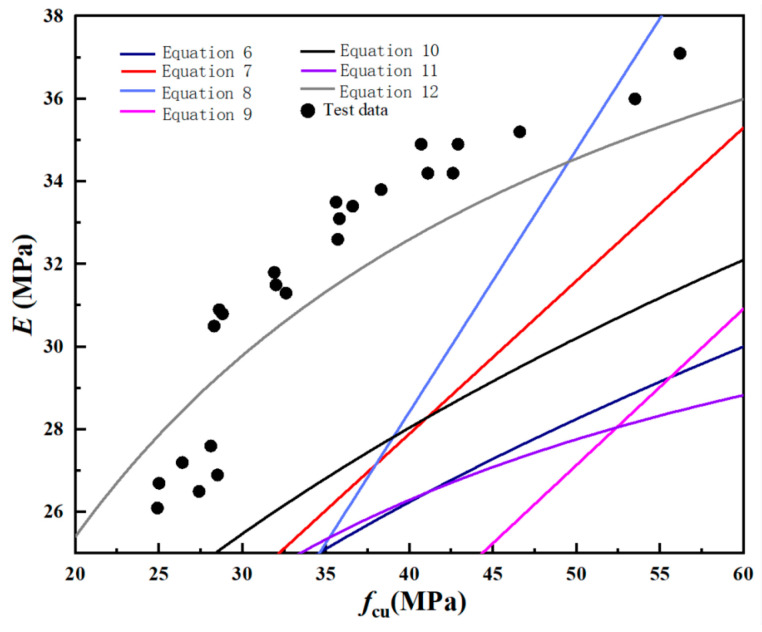
Theoretical Calculation Formula for Elastic Modulus of Recycled Concrete.

**Figure 7:**
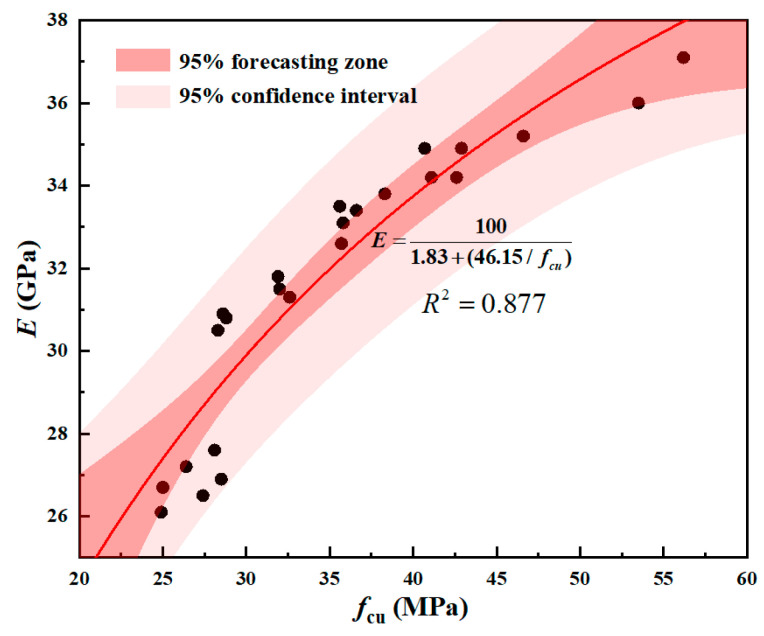
Regression Analysis Graph of Compressive Strength and Elastic Modulus of Mixed Coal Gangue.

As shown in Figure 6, both SCGA and RCGA concrete have a positive correlation between elastic modulus and compressive strength, with significant dispersion. Therefore, this paper reviews the formulas correlating the elastic modulus and compressive strength of recycled concrete from both domestic and international sources, seeking the model most suitable for this study. Table 8 shows the formulas for calculating the modulus of elasticity of recycled concrete proposed by different scholars from both domestic and foreign sources. Table 9 shows the theoretical modulus of elasticity calculated based on the formulas presented in Table 8 by different scholars from both domestic and foreign sources. Figure 7 shows the relationship between the theoretical modulus of elasticity calculation formulas and experimental values.

From Table 9 and Figure 6, it can be seen that the elastic modulus of coal gangue concrete calculated using the conventional concrete theoretical method Equation (12) yields results that are the closest. This indicates that this formula is relatively reliable for calculating the elastic modulus of coal gangue concrete based on the compressive strength of coal gangue. Therefore, appropriate modifications were made to Equation (12), as shown in Figure 7, resulting in Equation (13).

(13) $$E=\frac{10^{5}}{1.83+46.15/f_{cu}}$$

In the formula, *E* is the modulus of elasticity (MPa); *f_cu_* is the concrete strength (MPa).

**Figure 8:**
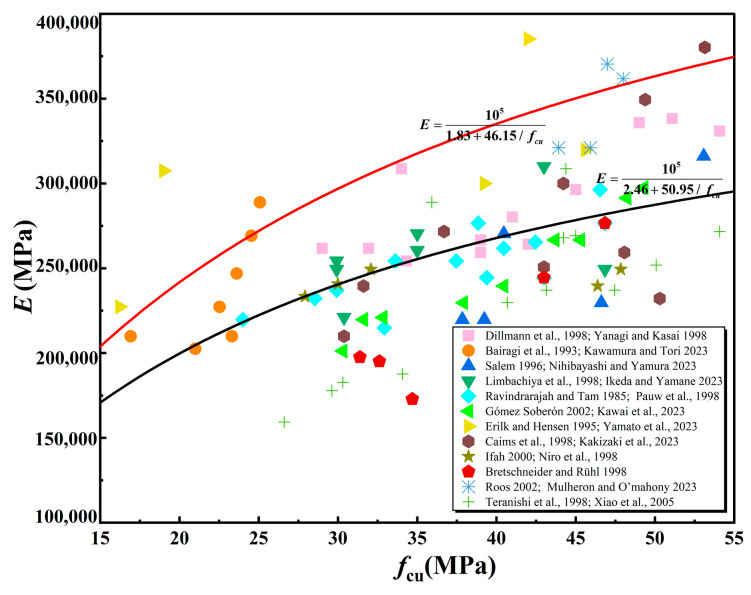
Equation (13) Adaptability in Recycled Concrete [45,47,51,52,53,54,55,56,57,58,59,60,61,62,63,64,65,66,67,68,69,70,71].

**Figure 9:**
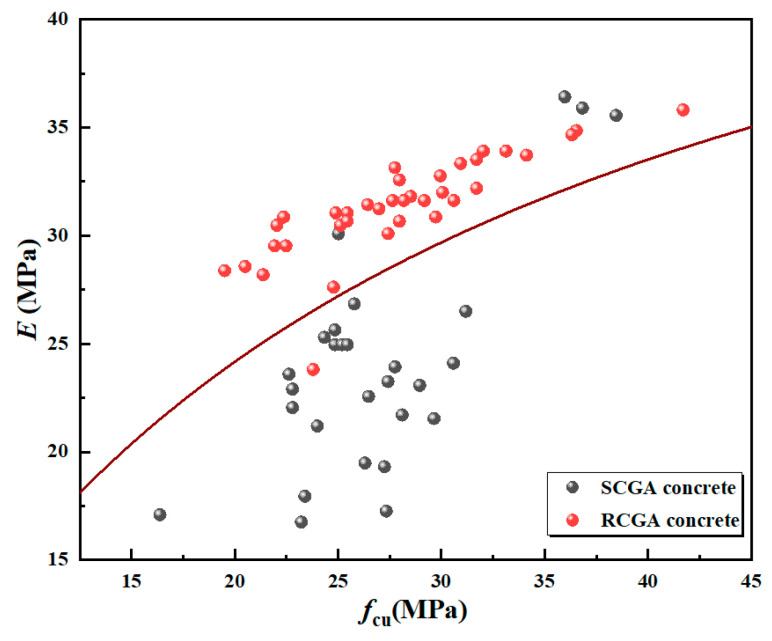
Equation (13) for SCRA and RCGA concrete.

Given that this research incorporates both SCGA and RCGA, it is essential to validate the predictive precision of the amended model presented in Equation (6). Data from 94 groups of experiments on the elastic modulus of recycled aggregate concrete collected from various researchers (Dillmann [47], Yanagi and Kasai [51], Bairagi et al. [52], Kawamura and Tori [53], Salem [54], Nihibayashi and Yamura [55], Limbachiya et al. [56], Ikeda and Yamane [57], Sri Ravindrarajah and Tam [45], De Pauw et al. [58], Gomez-Soberon [59], Kawai et al. [60], Erilk [61], Yamato et al. [62], Caims et al. [63], Kakizaki et al. [64], Ifah et al. [65], Di Niro et al. [66], Bretschneider and Ruhi [67], Roos [68], Mulheron and Omahony [69], Teranishi et al. [70], and Xiao et al. [71]) were summarized, as shown in Figure 8. Additionally, data from 28 groups of SCGA and 36 groups of RCGA concrete elastic modulus were summarized, as shown in Figure 9.

As shown in Figure 8, the corrected model Equation (13) overestimates the elastic modulus of recycled aggregate concrete. As shown in Figure 9, the corrected model Equation (13) overestimates the elastic modulus of natural concrete and underestimates the elastic modulus of non-natural concrete. This confirms that model Equation (13) accurately predicts the modulus of elasticity in mixed coal gangue concrete. Given that the compressive strength of synthetic concrete surpasses that of natural concrete, and considering the positive correlation between compressive strength and the modulus of elasticity, it follows that the modulus of elasticity for mixed concrete would be intermediate between the two. Hence, the modulus of elasticity of mixed concrete should be between the two. Regression fitting was performed on Figure 8, and it was found that the prediction model for the elastic modulus of recycled aggregate can be expressed as $E=\frac{10^{5}}{a+b/f_{cu}}$, where a and b are constants.

## 5. Interaction Mechanism of Mixed Coal Gangue Aggregate–Cement Paste Matrix

Coal gangue concrete is a porous, multi-scale, multiphase heterogeneous system. On a macroscopic scale, coal gangue concrete is a composite material consisting of cement paste, aggregate, and an interfacial transition zone. On a microscopic scale, the cement paste is a multiphase composite consisting of various hydrates, water, unhydrated particles, and air. Drawing from the distribution and enrichment of interfacial chemical elements, alongside the micromorphological characteristics of the interface, the interaction mechanisms between hardened cement mortar, the interfacial transition zone, and coarse aggregate can be categorized into three distinct types: physical action, physicochemical composite action, and mechanical interlocking action. The corresponding forces driving these interactions are van der Waals forces, chemical bonding, and mechanical engagement, respectively.

The coal gangue used in this study is a mixture of SCRA and RCGA. RCGA has a stable crystalline structure with low or no reactivity. It is generally considered that there are no conditions for chemical reactions between RCGA aggregate and cement paste. Its main effects are physical and mechanical actions involving van der Waals forces. Moreover, its multiple cavities capture significant moisture, impacting the mixture’s workability. In formulating low-grade concrete, the elevated water-to-cement ratio and the pronounced porosity (lower packing density) of RCGA lead to moisture absorption, diminishing the water-to-cement ratio and consequently enhancing hydration reactions. As the reaction progresses, the moisture absorbed by RCGA is slowly released, ensuring good humidity inside the concrete and providing the moisture needed for the hydration of the cementitious materials in the concrete. The hydration reaction will continue, allowing the later strength of the concrete to continue to grow. Therefore, when using coal gangue aggregate to configure C20 concrete, the 28-day compressive strength remains essentially unchanged or improved. Nevertheless, when high-grade cement is formulated, and the substitution rate attains a specific threshold, its excessive water absorption impairs hydration, particularly the initial strength. Concurrently, the intrinsic crushing value of coal gangue is insufficient and presents other shortcomings, failing to satisfy the demands for high-grade strength and, consequently, resulting in its diminution.

In SCGA, the concentrations of SiO_2_ and Al_2_O_3_ are elevated, classifying it as a volcanic ash material. It exhibits inherent pozzolanic activity, which enhances its cementitious properties. The reactive components of SiO_2_ and Al_2_O_3_ present in SCGA fine aggregates can participate in secondary hydration reactions, leading to the formation of novel compounds such as gismondine ((1): CaAl_2_Si_2_O_8_4H_2_O), hydrogarnet ((2): Ca_3_Al_2_(SiO_4_)_3_), and andradite ((3): Ca_3_(Fe_0.87_Al_0.13_)_2_(SiO_4_)_1.65_ (OH)_5.4_). These compounds significantly enhance the bonding at the interface between the aggregate and the cement matrix, thereby augmenting the compressive strength of the concrete [72]. SCGA affects the hydration of cement through two mechanisms: (1) high water absorption accelerates the hydration rate of cement, forming ettringite (3CaO·Al_2_O_3_·3CaSO_4_·32H_2_O) crystals; (2) the active silica and alumina constituents within coal gangue absorb the initial hydration products, such as Ca(OH)_2_, released during the hydration of cement, and engage in a subsequent hydration reaction with these compounds. This secondary hydration reaction within the cement consumes Ca(OH)_2_ crystals to produce ettringite crystals, effectively decreasing the quantity of Ca(OH)_2_ crystals. These secondary hydrates populate the cement paste framework and, in conjunction with the cement hydrates, form the matrix binder of the cement paste, thereby enhancing the density of the structure and augmenting both the microstructure and mechanical properties of the concrete. During the hydration process of coal gangue concrete, minerals such as potassium and sodium feldspar within the coal gangue break down in the presence of water, releasing soluble ions, including potassium, magnesium, and cadmium. As these decomposition products are predominantly alkaline, the resulting ions migrate to the hardened cement mortar, where they accumulate in the interfacial transition zone. Here, they form a gel characterized by high alkalinity and fine particles. This gel is particularly susceptible to drying, shrinkage, and cracking in arid conditions, thereby creating a fragile layer at the coal gangue–concrete interface that detrimentally impacts the structural integrity. Concurrently, the low crushing value and high porosity of the material remain unmitigated, culminating in compromised strength. The combined effect of SCGA’s secondary hydration reaction and RCGA’s strong water absorption leads to differences between the elastic modulus prediction model and previous elastic models.

Ca(OH)_2_ + Al_2_O_3_ + SiO_2_+ 4H_2_O → CaAl_2_Si_2_O_8_4H_2_O (14)

3CaAl_2_(SiO_4_) (OH)_2_ + Ca(OH)_2_ + CaCO_3_ → Ca_3_Al_2_(SiO_4_)_3_ + 4H_2_O + CO_2_↑ (15)

CaAl_2_Si_2_O_8_4H_2_O + 1.74Fe^3+^ → Ca(Fe_0.87_Al_0.13_)_2_(SiO_4_)_1.65_ (OH)_5.4_ + 2.6H^+^ (16)

C_3_A + 3CaSO_4_ + 32H_2_O → C_3_A·3CaSO_4_·32H_2_O (17)

## 6. Conclusions

This paper uses mixed coal gangue as coarse aggregate for concrete and conducts experimental studies and detailed discussions on the relationship between the mechanical properties of coal gangue concrete and the prediction model of elastic modulus, considering the strength grades of concrete (C20, C30, C40, C50) and substitution rates (0%, 20%, 40%, 60%, 80%, 100%). The main conclusions are as follows.

(1) Utilizing a blend of coal gangue as a substitute for coarse aggregate in the production of C20 strength concrete, it is observed that an increase in the coal gangue content does not markedly influence the concrete’s strength or its modulus of elasticity. However, substituting over 40% of the coarse aggregate in C30 strength concrete and more than 20% in C40 and C50 strength concrete significantly compromises both the modulus of elasticity and the compressive strength.
(2) The superior grade of concrete exhibits a marked reduction in elastic modulus and compressive strength in mixed gangue concrete as the aggregate substitution rate escalates. Notably, when the mixed gangue content surpasses 40%, the compressive strength of C40 and C50 concrete diminishes by 11% and 17%, respectively. Upon reaching 100% admixture, the strength of C50 concrete diminishes by 35%, significantly below the designated strength. At the same admixture level, the elastic modulus of C30 concrete decreases by 3.5%; furthermore, C40 and C50 concrete exhibit a notable decline in strength at a 40–60% admixture, with a maximum decrease of 6%, and at 100% admixture, the elastic modulus is reduced by 10%.
(3) Consider the correlation between the compressive strength and axial compressive strength of concrete with different coal gangue admixture contents and different concrete strength grades, and ordinary concrete. Establishing a regression equation between their mechanical properties will help in predicting and promoting the mechanical properties of coal gangue aggregate concrete.
(4) Drawing on the correlation between the modulus of elasticity and the compressive strength of SCGA and RCGA concrete, alongside seven established formulas for determining the modulus of elasticity in recycled concrete, a refined equation for the modulus of elasticity in mixed coal gangue concrete was developed: $E=\frac{10^{5}}{1.83+46.15/f_{cu}}$. This equation significantly enhances the accuracy of predictions. Moreover, following an analysis of 158 datasets, this predictive model was further optimized, culminating in a more robust formula for calculating the modulus of elasticity of recycled aggregate: $E=\frac{10^{5}}{a+b/f_{cu}}$.

## Figures and Tables

**Table 1:** Main Mineral Composition of Mixed Coal Gangue (%).

Materials	Quartz	Kaolin	Pyrite	Limestone	Mica
Mixed Coal Gangue	53.39	26.41	5.59	9.79	Minimal amount

**Table 2:** Main Chemical Components of Mixed Coal Gangue (%).

Materials	SiO_2_	Fe_2_O_3_	Al_2_O_3_	CaO	MgO	SO_3_
Mixed Coal Gangue	42.31	25.69	23.28	2.69	0.7	0.69

**Table 3:** Technical Properties of Mixed Coal Gangue. (According to GB/T14685-2010-11 Construction Pebbles and Crushed Stone).

Diameter (mm)	Bulk Density (kg/m^3^)	Apparent Density (g/cm^3^)	Water Absorption (%)	Crushing Index (%)
5–25	1380	2.60	4.4	12.6

**Table 4:** Technical properties of coarse aggregates. (According to GB/T14685-2010-11 Construction Pebbles and Crushed Stone).

Diameter (mm)	Bulk Density (kg/m^3^)	Apparent Density (g/cm^3^)	Water Absorption (%)	Crushing Index (%)
5–25	1560	2.72	1.1	11.2

**Table 5:** Concrete Mix Proportions [20].

Strength Grade	Sand Content (%)	W/Binder	Cement	Fly Ash	W	Sand	Mixed Coal Gangue	Cobble	Water Reducer	Silica Fume
C20	41	0.5	280	80	180	763	1097	0	3.5	—
	41	0.5	280	80	180	763	878	219	2.9	—
	41	0.5	280	80	180	763	658	439	2.7	—
	41	0.5	280	80	180	763	439	658	2.5	—
	41	0.5	280	80	180	763	219	878	2.3	—
	41	0.5	280	80	180	763	0	1097	2.1	—
C30	40	0.4	347	100	180	721	1082	0	3.2	—
	40	0.4	347	100	180	721	866	216	2.8	—
	40	0.4	347	100	180	721	649	433	2.6	—
	40	0.4	347	100	180	721	433	649	2.4	—
	40	0.4	347	100	180	721	216	866	2.2	—
	40	0.4	347	100	180	721	0	1082	2.0	—
C40	39	0.35	412	80	170	698	1076	0	5.2	—
	39	0.35	412	80	170	698	857	215	3.0	—
	39	0.35	412	80	170	698	644	430	2.8	—
	39	0.35	412	80	170	698	430	644	2.6	—
	39	0.35	412	80	170	698	215	857	2.4	—
	39	0.35	412	80	170	698	0	1076	2.2	—
C50	38	0.31	450	70	165	671	1070	0	4.0	18
	38	0.31	450	70	165	671	856	214	3.3	18
	38	0.31	450	70	165	671	640	428	3.1	18
	38	0.31	450	70	165	671	428	642	2.9	18
	38	0.31	450	70	165	671	214	856	2.7	18
	38	0.31	450	70	165	671	0	1067	2.5	18

**Table 6:** Mechanical Properties of Coal Gangue Concrete [20].

Strength Grade	Mixed Coal Gangue Content (%)	Compressive Strength of Cube/*f_cu_* (MPa)	Axial Compressive Strength/*f_ck_* (MPa)	Modulus of Elasticity/E (GPa)
C20	100	26.4	15.85	27.2
	80	28.5	17.04	26.9
	60	25.0	17.66	26.7
	40	27.4	19.53	26.5
	20	28.1	19.15	27.6
	0	24.9	17.33	26.1
C30	100	28.3	17.08	30.5
	80	28.8	16.59	30.8
	60	28.6	18.74	30.9
	40	32.6	19.69	31.3
	20	32.0	20.02	31.5
	0	31.9	22.58	31.8
C40	100	35.7	15.37	32.6
	80	35.8	16.59	33.1
	60	35.6	17.06	33.5
	40	38.3	20.67	33.8
	20	41.1	28.18	34.2
	0	42.9	30.75	34.9
C50	100	36.6	16.38	33.4
	80	42.6	18.31	34.2
	60	40.7	18.84	34.9
	40	46.6	24.07	35.2
	20	53.5	37.57	36.0
	0	56.2	39.75	37.1

**Table 7:** Analysis Results of Axial Compressive Strength of Coal Gangue Concrete.

Strength Grade	Mixed Coal Gangue Content (%)	*f_cu_* (MPa)	*f_ck_* (MPa)	*f_c_* (MPa)	Yes/No	*f_c_*/*f_cu_*
C20	100	26.4	13.4	15.85	Yes	0.60
	80	28.5		17.04	Yes	0.60
	60	25		17.66	Yes	0.71
	40	27.4		19.53	Yes	0.71
	20	28.1		19.15	Yes	0.68
	0	24.9		17.33	Yes	0.70
C30	100	28.3	20.1	17.08	No	0.60
	80	28.8		16.59	No	0.58
	60	28.6		18.74	No	0.66
	40	32.6		19.69	No	0.60
	20	32		20.02	Yes	0.63
	0	31.9		22.58	Yes	0.71
C40	100	35.7	26.8	15.37	No	0.43
	80	35.8		16.59	No	0.46
	60	35.6		17.06	No	0.48
	40	38.3		20.67	No	0.54
	20	41.1		28.18	Yes	0.69
	0	42.9		30.75	Yes	0.72
C50	100	36.6	32.4	16.38	No	0.45
	80	42.6		18.31	No	0.43
	60	40.7		18.84	No	0.46
	40	46.6		24.07	No	0.52
	20	53.5		37.57	Yes	0.70
	0	56.2		39.75	Yes	0.71

Note: *f_ck_* is the standard for axial compressive strength of normal concrete.

**Table 8:** Theoretical Calculation Model of Elastic Modulus of Recycled Concrete.

Model	Expression	Proposer
Equation (6)	*E* = 7770*f_cu_*^0.33^	Sri Ravindrarajah et al. [45]
Equation (7)	*E* = 370*f_cu_* + 13100	Dhir et al. [46]
Equation (8)	*E* = 634.43*f_cu_* + 3057.6	Dillmann et al. [47]
Equation (9)	*E* = 378*f_cu_* + 8242	Mellmann et al. [48]
Equation (10)	$E=18800\sqrt[{3}]{\frac{0.83f_{cu}}{10}}$	Corinaldesi et al. [49]
Equation (11)	$E=\frac{10^{5}}{2.8+40.1/f_{cu}}$	Xiao et al. [50]
Equation (12)	$E=\frac{10^{5}}{2.2+34.7/f_{cu}}$	GB 50010-2002

In the formula, *E* is the modulus of elasticity (MPa); *f_cu_* is the concrete strength (MPa).

**Table 9:** Experimental and Theoretical Values of Elastic Modulus of Mixed Coal Gangue Concrete.

Strength Grade	Mixed Coal Gangue Content (%)	Test Value (GPa)	Equation (6): E (GPa)	Equation (7): E (GPa)	Equation (8): E (GPa)	Equation (9): E (GPa)	Equation (10): E (GPa)	Equation (11): E (GPa)	Equation (12): E (GPa)
C20	100	27.2	22.77	22.87	19.81	18.22	24.42	26.89	28.45
	80	26.9	23.35	23.65	21.14	19.02	25.05	27.72	29.26
	60	26.7	22.36	22.35	18.92	17.69	23.98	26.29	27.87
	40	26.5	23.05	23.24	20.44	18.60	24.72	27.30	28.85
	20	27.6	23.24	23.50	20.89	18.86	24.93	27.57	29.11
	0	26.1	22.33	22.31	18.85	17.65	23.95	26.24	27.83
C30	100	30.5	23.30	23.57	21.01	18.94	24.99	27.65	29.19
	80	30.8	23.43	23.76	21.33	19.13	25.14	27.84	29.37
	60	30.9	23.38	23.68	21.20	19.05	25.08	27.76	29.30
	40	31.3	24.41	25.16	23.74	20.56	26.20	29.15	30.63
	20	31.5	24.26	24.94	23.36	20.34	26.04	28.96	30.45
	0	31.8	24.23	24.90	23.30	20.30	26.01	28.93	30.42
C40	100	32.6	25.15	26.31	25.71	21.74	27.00	30.09	31.53
	80	33.1	25.17	26.35	25.77	21.77	27.03	30.12	31.55
	60	33.5	25.13	26.27	25.64	21.70	26.98	30.06	31.50
	40	33.8	25.74	27.27	27.36	22.72	27.64	30.80	32.20
	20	34.2	26.35	28.31	29.13	23.78	28.30	31.49	32.85
	0	34.9	26.72	28.97	30.27	24.46	28.71	31.90	33.24
C50	100	33.4	25.36	26.64	26.28	22.08	27.23	30.34	31.77
	80	34.2	26.66	28.86	30.08	24.34	28.64	31.83	33.17
	60	34.9	26.26	28.16	28.88	23.63	28.21	31.39	32.76
	40	35.2	27.46	30.34	32.62	25.86	29.51	32.67	33.96
	20	36	28.74	32.90	37.00	28.47	30.90	33.90	35.10
	0	37.1	29.21	33.89	38.71	29.49	31.41	34.32	35.49

## Data Availability

The original contributions presented in this study are included in the article. Further inquiries can be directed to the corresponding authors.
